# Luminescent Properties and Optical Temperature Sensing Performance of CaTa_2_O_6_:Pr^3+^ Phosphors Under Blue-Light Excitation

**DOI:** 10.3390/ma19112324

**Published:** 2026-06-01

**Authors:** Quan Jiang, Jian Ruan, Chen Tian, Zijing Zhu, Shuang Zhang, Chao Liu

**Affiliations:** 1 State Key Laboratory of Advanced Glass Materials, Wuhan University of Technology, Wuhan 430070, China; quanjiang_0382@whut.edu.cn (Q.J.); zijing@whut.edu.cn (Z.Z.); shuangzhang_22@whut.edu.cn (S.Z.); hite@whut.edu.cn (C.L.); 2 Research Center for Silicate Materials Engineering, Wuhan University of Technology, Wuhan 430070, China; 3 State Key Laboratory of Silicate Materials for Architectures, Wuhan University of Technology, Wuhan 430070, China

**Keywords:** Pr^3+^, CaTa_2_O_6_ phosphor, optical temperature sensing, fluorescence intensity ratio, IVCT state, phosphor-in-glass

## Abstract

**Highlights:**

- The concentration-dependent luminescence of CaTa_2_O_6_:Pr^3+^ phosphors under blue-light excitation is presented.
- Emission enhancement via Pr^3+^/Zr^4+^ or Pr^3+^/Sn^4+^ co-doping is found.
- The temperature-dependent luminescence of the CaTa_2_O_6_:Pr^3+^, Sn^4+^ phosphor and a PiG based on it are studied.
- The FIR mode based on ^3^P_1_→^3^H_5_/^3^P_0_→^3^F_2_ of Pr^3+^ is found to be promising for luminescence thermometry.

**Abstract:**

Pr^3+^-activated phosphors are promising for non-contact optical thermometry under blue-light excitation. In tantalate hosts, Pr^3+^-Ta^5+^ intervalence charge transfer (IVCT) states may introduce thermally activated nonradiative pathways involving the ^3^P_0_ and ^1^D_2_ levels, thus affecting their thermal quenching behavior and thermometric performance. However, the concentration- and temperature-dependent luminescence of CaTa_2_O_6_:Pr^3+^ remains unexplored. In this study, CaTa_2_O_6_:Pr^3+^ phosphors were synthesized via the solid-state reaction method, and a phosphor-in-glass (PiG) composite was fabricated by co-sintering the mixture of the phosphor and the precursor glass (PG) powder. The structural characteristics and the luminescence properties of CaTa_2_O_6_:Pr^3+^ phosphors under 450 nm excitation were investigated. The IVCT band was confirmed in the excitation spectrum. Optimal Pr^3+^ concentrations were 2 mol% for ^3^P_J_ and 0.7 mol% for ^1^D_2_ emissions. With Pr^3+^/Zr^4+^ or Pr^3+^/Sn^4+^ co-doping, the emission intensity was enhanced by 1.34 and 1.31 times, respectively. The PiG exhibited similar spectral profiles. An FIR mode based on ^3^P_1_→^3^H_5_/^3^P_0_→^3^F_2_ transitions achieved maximum relative sensitivities of 1.09% K^−1^ for the phosphor and 1.18% K^−1^ for the PiG at 298 K. These findings suggest that CaTa_2_O_6_:Pr^3+^-based materials are potential candidates for luminescence thermometry.

## 1. Introduction

Pr^3+^-activated inorganic phosphors are promising for optical sensing because of their abundant energy levels in the 4f^2^ electronic configuration and excellent luminescence properties [1,2]. Luminescence thermometry has attracted considerable attention because of its non-contact nature, rapid response, and strong resistance to electromagnetic interference [3,4]. The fluorescence intensity ratio (FIR) technique is particularly attractive because of its self-referencing feature, which minimizes the effects of excitation fluctuations and improves measurement reliability [5]. In particular, the thermally coupled ^3^P_1_/^3^P_0_ levels of Pr^3+^ ions enable a typical Boltzmann-type temperature response. Moreover, in oxide hosts containing d^0^ cations, such as Ti^4+^, Nb^5+^ or Ta^5+^, the luminescence of Pr^3+^ is frequently affected by intervalence charge transfer (IVCT) states. It provides an efficient nonradiative channel for electron relaxing from excited state ^3^P_0_ to excited state ^1^D_2_ due to its strongly temperature-dependent behavior, so that the IVCT process can significantly improve optical thermometric performance [6,7,8,9].

Recently, CaTa_2_O_6_ has been proven to be a promising host for rare-earth ion activators because of its high stability [10,11,12,13]. The presence of Ta^5+^ provides favorable conditions for the formation of Pr^3+^-Ta^5+^-related IVCT states. CaTa_2_O_6_:Pr^3+^ has been reported to exhibit unusual greenish blue emission with a pronounced contribution from the ^3^P_0_ level, suggesting that the IVCT state in this host is not sufficiently effective to fully quench the ^3^P_0_ emission. In addition, multiple electron trapping centers have been identified in CaTa_2_O_6_:Pr^3+^, indicating that defect-assisted processes also contribute to its luminescence and thermal relaxation behavior [14]. Therefore, the luminescence of Pr^3+^ in CaTa_2_O_6_ is governed by the combined effects of intrinsic 4f-4f transitions, IVCT-assisted relaxation, and defect-related nonradiative processes, resulting in temperature-dependent behavior distinct from that of conventional thermally coupled-level systems [15,16]. Nevertheless, the concentration- and temperature-dependent luminescence of CaTa_2_O_6_:Pr^3+^ under blue-light excitation remains unexplored, but this is significant in understanding the thermal sensitivity of its luminescence and assessing its potential for optical thermometry.

Moreover, Pr^3+^-activated phosphors could benefit efficient excitation via commercially available blue-light sources in the 440–470 nm range [17]. This makes them favorable for compact and low-cost non-contact thermometric devices. Furthermore, a phosphor-in-glass (PiG) composite approach would allow it to be used as a fiber-optic probe material. Because the inorganic glass matrix offers superior thermal stability, moisture resistance, and long-time durability compared with conventional silicone or resin packing, the PiG material would be promising for fiber-integrated optical thermometry [18].

Therefore, in this study, the photoluminescence properties of CaTa_2_O_6_:Pr^3+^ phosphors under 450 nm excitation are reported. Their phase structures and morphologies were examined. The Pr^3+^ concentration-dependent luminescence and the effect of charge compensation by Sn^4+^ and Zr^4+^ co-doping were investigated. Furthermore, a PiG sample combining the CaTa_2_O_6_:0.02Pr, 0.02Sn phosphor and the 30ZnO-30Bi_2_O_3_-40B_2_O_3_ glass was fabricated. The temperature-dependent luminescence and optical thermometric performance of the phosphor and the PiG based on it were evaluated. The results are expected to provide useful guidance for the development of Pr^3+^-activated tantalate luminescent thermometric materials and related PiG devices.

## 2. Materials and Methods

The conventional high-temperature solid-state reaction method was used for preparing Ca_1−*x*_Ta_2_O_6_:*x*Pr^3+^ (*x* = 0.005–0.05) and charge-compensated Ca_0.98_Ta_1.98_O_6_:0.02Pr^3+^, 0.02M^4+^ (M = Zr and Sn) phosphors. Stoichiometric amounts of CaCO_3_ (99.9%), Ta_2_O_5_ (99.5%), Pr_6_O_11_ (99.0%), SnO (99.0%), and ZrO_2_ (99.0%) were thoroughly mixed and ground and then calcined in alumina crucibles at 800 °C for 1 h and 1200 °C for 6 h, followed by cooling to room temperature. Then, 3 wt% H_3_BO_3_ (99.0%) was added as a flux for sintering. The obtained products were crushed and reground into powders. For simplicity, all CaTa_2_O_6_-based phosphors are denoted as CTO:*x*Pr, CTO:0.02Pr, 0.02Sn, or CTO:0.02Pr, 0.02Zr hereafter. Low-melting glass powders were prepared via the traditional melt-quenching route. The nominal chemical composition of the precursor glass (PG) is 30ZnO-30Bi_2_O_3_-40B_2_O_3_ (in mol%), denoted as 30Zn30Bi40B. The raw materials of ZnO (99.0%), Bi_2_O_3_ (99.0%), and H_3_BO_3_ (99.0%) were mixed and melted at 800 °C for 1 h to obtain homogeneous glass melts. The glass was formed in a stainless-steel mold and then cooled to room temperature. Then, it was crushed and ground into powders. The PiG samples were fabricated from the mixture of the CaTa_2_O_6_:0.02Pr, 0.02Sn phosphor and the 30Zn30Bi40B glass powder with a weight ratio of 1:9. After being thoroughly ground, the phosphor–glass powder mixture was pressed into a disk-shaped pellet and then co-sintered in a muffle furnace at 480 °C for 1 h. After cooling to room temperature, the PiG samples were obtained.

The phase compositions of both the phosphor samples and the PiG sample were characterized by X-ray diffraction (XRD, Bruker D8 Advance, Cu Kα radiation) in the 2θ range of 10–80° with a step size of 0.02°, and the phases were identified by comparison with standard PDF cards. The microstructure and elemental distribution were investigated by field-emission scanning electron microscopy (FE-SEM, Zeiss Ultra Plus) coupled with energy-dispersive X-ray spectroscopy (EDS, X-Max50). For the PiG sample, EDS analysis was performed to distinguish the glass and crystalline phases and to evaluate their interfacial features. The thermal behavior of the PG was studied by differential scanning calorimetry (DSC, STA449F3) under flowing Ar at a heating rate of 10 °C min^−1^. The diffuse reflectance spectra of the phosphors and transmittance spectra of the PG were measured with a UV–vis–NIR spectrophotometer (Lambda 750 S). Photoluminescence excitation and emission spectra were recorded on a PTI QM/TM/NIR spectrofluorometer using an MDL-III-450 laser as the excitation source, with a step size of 1 nm and an integration time of 0.1 s. Temperature-dependent luminescence measurements were carried out on the same system fitted with a TAP-02 high-temperature fluorescence controller over 298–573 K, using a temperature interval of 25 K and a holding time of 5 min at each temperature.

## 3. Results and Discussion

### 3.1. CaTa_2_O_6_:Pr^3+^ Phosphors

#### 3.1.1. Phase Structure and Morphology

**Figure 1:**
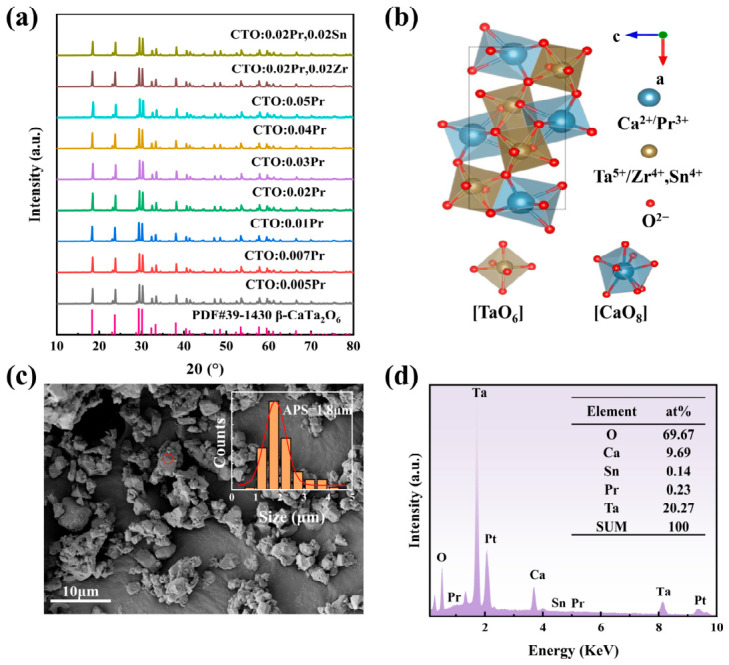
(**a**) XRD patterns of CTO: Pr^3+^ phosphors with various Pr^3+^ doping concentrations and different charge compensators; (**b**) crystal structure of β-CaTa_2_O_6_; (**c**) SEM image of the CTO:0.02Pr, 0.02Sn phosphor, the red circle marks the position selected for EDS analysis, and the inset shows the particle size distribution; (**d**) EDS spectrum of the CTO:0.02Pr, 0.02Sn phosphor.

The XRD patterns of the CaTa_2_O_6_:Pr^3+^ phosphors are given in Figure 1a. It can be seen that all diffraction peaks could be well indexed to the standard pattern of β-CaTa_2_O_6_. This suggests that the β-CaTa_2_O_6_ phase was successfully obtained in CTO:*x*Pr (*x* = 0.005–0.05), CTO:0.02Pr, 0.02Sn, and CTO:0.02Pr, 0.02Zr samples. No obvious peaks from secondary phases were observed. These results indicate that the CTO host exhibits good structural tolerance toward Pr^3+^ doping and charge compensation.

Figure 1b illustrates the crystal structure of β-CaTa_2_O_6_ (space group Pnma, No. 62), in which Ca^2+^ occupies an eight-coordinated site and Ta^5+^ occupies a six-coordinated site. Based on the Shannon ionic radii [19], Pr^3+^ (1.13 Å, CN = 8) is expected to substitute Ca^2+^ (1.12 Å, CN = 8) owing to their comparable sizes. In contrast, Zr^4+^ (0.72 Å, CN = 6) and Sn^4+^ (0.69 Å, CN = 6) exhibit ionic radii similar to that of Ta^5+^ (0.64 Å, CN = 6) under six-fold coordination, suggesting the preferential occupancy of the Ta sites. Such site-selective doping helps to preserve the structural stability of the β-CaTa_2_O_6_ host.

As shown in Figure 1c, irregular particles with an average size of 1.8 μm were observed in the CTO:0.02Pr,0.02Sn sample. Slight particle agglomeration was also found, which was likely caused by particle coalescence and grain growth during high-temperature sintering [20]. It suggests that the phosphor should be suitable for PiG fabrication. Moreover, the presence of Ca, Ta, O, Pr, and Sn was confirmed by EDS analysis. As given in Figure 1d, the atomic ratio of Ca to Ta is close to 1:2, in good agreement with the stoichiometry of CaTa_2_O_6_, while the slightly reduced Ca content can be ascribed to the partial substitution of Ca by Pr and Sn.

#### 3.1.2. Photoluminescence Properties

**Figure 2:**
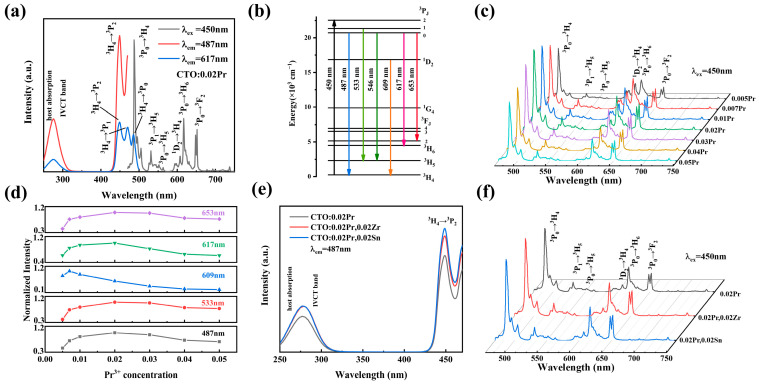
(**a**) Excitation and emission spectra of the CTO:0.02Pr sample; (**b**) schematic energy-level diagram of CTO:Pr^3+^; (**c**) emission spectra of CTO:*x*Pr (*x* = 0.005–0.05) under 450 nm excitation; (**d**) normalized integrated emission intensities of different emission bands marked by the wavelength at the intensity maximum; (**e**) excitation and (**f**) emission spectra of CTO:0.02Pr and CTO:0.02Pr, 0.02M (M = Zr and Sn).

As shown in Figure 2a, characteristic transitions of Pr^3+^ ions were observed in the excitation spectra and the emission spectrum of CTO:0.02Pr. When monitored at 487 and 617 nm, respectively, the excitation spectra were similar in shape, indicating that both emissions originated from the same Pr^3+^ center. Typical excitation bands contributing to the 4f-4f transitions of Pr^3+^ were observed at 450 nm, 470 nm, and 486 nm. A broad excitation band in the UV region was also found, which could be assigned to the overlapping of the host-related absorption and IVCT bands. Under 450 nm excitation, the strongest emission band was found at 487 nm. The intense cyan–greenish emission could be ascribed to the ^3^P_0_→^3^H_4_ transition of Pr^3+^. Moreover, the sample also exhibited other characteristic emissions of Pr^3+^, including ^3^P_1_→^3^H_5_, ^3^P_0_→^3^H_5_, ^1^D_2_→^3^H_4_, ^3^P_0_→^3^H_6_, and ^3^P_0_→^3^F_2_. The corresponding schematic energy-level diagram of Pr^3+^ in CTO is illustrated in Figure 2b. There is obvious splitting in the emission bands of ^3^P_0_→^3^H_4_, ^1^D_2_→^3^H_4_, ^3^P_0_→^3^H_6_, and ^3^P_0_→^3^F_2_, suggesting that Pr^3+^ occupies a low-symmetry local environment in the CTO lattice.

The emission spectra of CTO phosphors varying with Pr^3+^ concentrations are shown in Figure 2c. Similar spectral profiles are observed in them, indicating that the luminescence centers and their local environments remained essentially unchanged within the investigated concentration range. The concentration-dependent emission intensity is summarized in Figure 2d. The concentration-induced quenching at the ^1^D_2_ level (0.7 mol%) was found to occur at a lower Pr^3+^ doping concentration compared to that (2 mol%) at the ^3^P_J_ levels (J = 1 or 0). This phenomenon could be explained by their different responses induced by the energy migration and cross-relaxation processes [21,22,23]. The CIE chromaticity coordinates of the CTO phosphors with different Pr^3+^ concentrations are shown in Figure S1 and Table S1. With increasing Pr^3+^ concentrations, the emission color gradually shifts from the orange–red region ((x, y) = (0.436, 0.370)) toward the yellow–green region ((x, y) = (0.326, 0.399)). This behavior is consistent with the different concentration quenching characteristics of the ^1^D_2_ and ^3^P_J_ levels.

The excitation and emission spectra of the CTO:0.02Pr and CTO:0.02Pr, 0.02M (M = Zr and Sn) phosphors are shown in Figure 2e and Figure 2f, respectively. In comparison with the singly Pr^3+^-doped samples, there was apparently stronger emission in the samples with Pr^3+^/Zr^4+^ or Pr^3+^/Sn^4+^ co-doping under 450 nm excitation. The emission intensity in CTO:0.02Pr, 0.02Zr, and CTO:0.02Pr, 0.02Sn was 1.31 times and 1.34 times that in CTO:0.02Pr, respectively. The enhancement could mainly be ascribed to improved charge compensation, which suppresses the defect-related nonradiative relaxation induced by the substitution of Ca^2+^ by Pr^3+^ [24]. In addition, charge compensation might also contribute to the stabilization of the local coordination environment around Pr^3+^ [25]. The better performance of the sample co-doped with Sn could further be related to possible valence regulation during sintering, leading to an increased population of effective Pr^3+^ luminescence centers.

#### 3.1.3. Temperature-Dependent Photoluminescence and Optical Thermometric Performance

**Figure 3:**
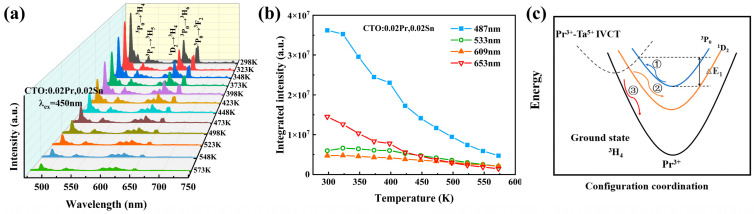
(**a**) Temperature-dependent emission spectra of the CTO:0.02Pr,0.02Sn phosphor; (**b**) the integrated intensities of different emission bands marked by the wavelength at the intensity maximum; (**c**) configuration coordinate diagram of the nonradiative relaxation processes of Pr^3+^during heating: ① ^3^P_0_→IVCT; ② IVCT→^1^D_2_; ③ IVCT→^3^H_4_.

The emission spectra at different environmental temperatures from 298 to 573 K were measured in the CTO:0.02Pr, 0.02Sn sample under 450 nm excitation, as shown in Figure 3a. It was selected because it exhibited the most intense emission among all obtained samples. Most emissions were found to be quenched with the rising of the temperature. Figure 3b shows the temperature-dependent integrated intensities of four characteristic emission bands at 487, 533, 609, and 653 nm, corresponding to the transitions of ^3^P_0_→^3^H_4_, ^3^P_1_→^3^H_5_, ^1^D_2_→^3^H_4_, and ^3^P_0_→^3^F_2_, respectively. It can be seen that there was more significant thermal quenching in the ^3^P_0_-related emissions as compared to the ^3^P_1_- or ^1^D_2_-related emissions. The CIE chromaticity coordinates at different temperatures are shown in Figure S2 and Table S2. With the increasing temperature, the emission color of CTO:0.02Pr, 0.02Sn gradually shifted toward the orange–red region.

The thermal quenching at the ^3^P_0_ level is affected not only by the thermally coupled levels but also by nonradiative processes associated with the ^3^P_0_ and ^1^D_2_ levels. Previous studies have shown that the thermal quenching behavior at this level is strongly affected by the IVCT state [26,27,28,29,30,31]. A schematic diagram is provided in Figure 3c for better explanation. When excited at 450 nm, the electrons in the ground ^3^H_4_ state are firstly populated to the ^3^P_0_ excited state. Then, electrons located at the ^3^P_0_ excited state can acquire sufficient energy from thermal phonons with increasing temperatures. It overcomes the energy barrier Δ*E*_1_ and transfer to the Pr^3+^-Ta^5+^ IVCT state (process ①). Upon reaching the IVCT state, the electrons tend to relax toward lower-lying levels. Since the energy barrier between the IVCT state and the ^3^P_0_ level is significantly higher than that between the IVCT state and the ^1^D_2_ level, the electrons more readily relax to the ^1^D_2_ level (process ②) rather than returning to the ^3^P_0_ level, and some of them further relax to the ^3^H_4_ ground state (process ③). As a result, the population of the ^3^P_0_ level would continuously decrease with the rising temperature, leading to the pronounced thermal quenching of the ^3^P_0_-related emissions at high temperatures. In contrast, the ^1^D_2_ level can be partially replenished by the relaxation process from the ^3^P_0_ level and therefore exhibits comparatively weaker thermal quenching.

Since different emission bands exhibit different temperature dependences, suitable emission pairs can be further selected to construct the FIR parameter. To evaluate the thermometric performance of the CTO:0.02Pr, 0.02Sn phosphor, three FIR modes were employed as temperature-sensitive signals. They were ^3^P_1_→^3^H_5_/^3^P_0_→^3^H_4_, ^3^P_1_→^3^H_5_/^3^P_0_→^3^F_2_, and ^1^D_2_→^3^H_4_/^3^P_0_→^3^H_4_. The FIR–temperature relationship was fitted using the following equation [32]:

(1) $$\begin{array}{c} FIR=Aexp\left(-\frac{∆E}{k_{B}T}\right)+C=Aexp\left(-\frac{B}{T}\right)+C \end{array}$$

where Δ*E*, *T*, and k_B_ represent the energy difference, absolute temperature, and Boltzmann constant, respectively. *A* and *C* are the fitted curve’s relevant constant parameters, with *A* associated with the spontaneous emission rate, degeneracy, and angular frequency. Δ*E*/k_B_ is denoted as *B* to simplify the formula. The absolute sensitivity (*S*_a_) and relative sensitivity (*S*_r_) can be further derived as follows:

(2) $$\begin{array}{c} S_{a}=\left|\frac{dFIR}{dT}\right|=\frac{B}{T^{2}}\times A\times exp\left(-\frac{B}{T}\right) \end{array}$$

(3) $$\begin{array}{c} S_{r}=\left|\frac{1}{FIR}\frac{dFIR}{dT}\right|=\frac{B}{T^{2}}\times \frac{Aexp\left(-\frac{B}{T}\right)}{Aexp\left(-\frac{B}{T}\right)+C} \end{array}$$

**Figure 4:**
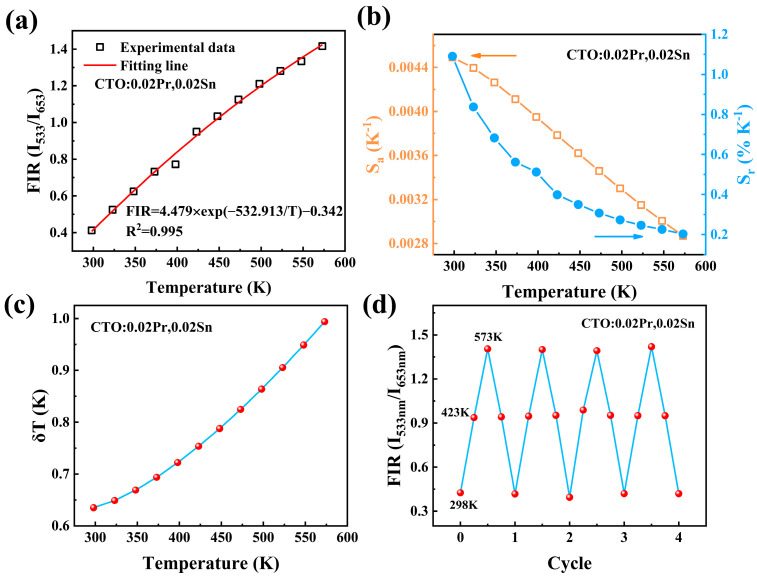
(**a**) FIR fitting curve; (**b**) the corresponding relative and absolute sensitivity curves; (**c**) temperature uncertainty (*δT*) as a function of temperature; and (**d**) temperature cycling of FIR between 298 and 573 K in the CTO:0.02Pr, 0.02Sn phosphor.

As shown in Figure 4a, in the CTO:0.02Pr, 0.02Sn phosphor, the FIR based on ^3^P_1_→^3^H_5_/^3^P_0_→^3^F_2_ was well fitted as a function of the temperature. As shown in Figure 4b, it exhibits maximum relative sensitivity of 1.09% K^−1^ at 298 K. This is much higher than that of the other two FIR modes based on ^3^P_1_→^3^H_5_/^3^P_0_→^3^H_4_ and ^1^D_2_→^3^H_4_/^3^P_0_→^3^H_4_, as presented in Figure S3. Moreover, temperature resolution (*δT*) is an important criterion for evaluating the sensing performance of optical thermometers. It is defined as the minimum temperature resolvable by the thermometer, depending not only on the material but also on the experimental environment used, such as the detection setup, acquisition conditions, and signal-to-noise ratio. Temperature resolution is given by the following equation [12]:

(4) $$\begin{array}{c} \delta T=∆T=\frac{\delta FIR}{S_{a}}=\frac{1}{S_{r}}\frac{\delta FIR}{FIR} \end{array}$$

where *δFIR* is the standard deviation of *FIR*. *δFIR*/*FIR* is the relative error, which is determined by multiple emission spectral measurements at a single temperature. Here, the emission spectra were measured ten times at room temperature to calculate the relative error for the entire temperature range. The temperature resolution *δT* for the FIR of ^3^P_1_→^3^H_5_/^3^P_0_→^3^F_2_ at 298–573 K was estimated to be 0.64–0.99 K, as shown in Figure 4c. Then, heating–cooling cycle measurements were carried out, and the corresponding FIR values are presented in Figure 4d. The reproducibility *R* was calculated according to the following equation:

(5) $$\begin{array}{c} R=1-\max\left|\frac{{FIR}_{i}-{FIR}_{av}}{{FIR}_{av}}\right| \end{array}$$

The reproducibility *R* reached 98.8%, 98.4%, and 98.9% at 298, 423, and 573 K, respectively, indicating that the FIR response remains highly stable and reproducible during thermal cycling.

### 3.2. CaTa_2_O_6_:Pr^3+^ PiG

#### 3.2.1. Phase Structure and Morphology

**Figure 5:**
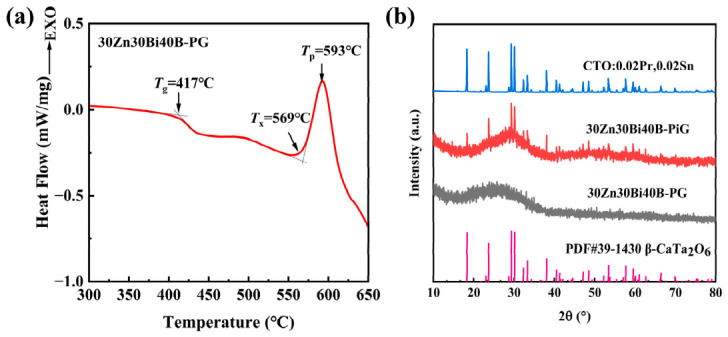
(**a**) DSC curve of 30Zn30Bi40B-PG; (**b**) XRD patterns of the PG, CTO:0.02Pr,0.02Sn phosphor, and corresponding PiG sample.

Figure 5a shows the DSC curve of 30Zn30Bi40B-PG, from which the glass transition temperature (*T*_g_), crystallization onset temperature (*T*_x_), and crystallization peak temperature (*T*_p_) were determined to be 417, 569, and 593 °C, respectively. The characteristic temperatures are low enough to ensure an acceptable softening point. The 30Zn30Bi40B glass is expected to offer good thermal processability, thereby minimizing the risk of phosphor degradation and interfacial reactions during co-sintering.

Figure 5b shows the XRD patterns of the PG, CTO:0.02Pr, 0.02Sn phosphor and the PiG sample. The PG exhibits a typical non-crystalline profile, in agreement with the nature of its amorphous structure. The phosphor sample shows sharp diffraction peaks, which matches well with the standard pattern of β-CaTa_2_O_6_ (PDF#39-1430). In the PiG sample, the characteristic diffraction peaks of β-CaTa_2_O_6_ with a background of non-crystallinity were observed. No extra diffraction peaks assignable to impurity phases were detected. This suggests that the phosphor was successfully incorporated.

**Figure 6:**
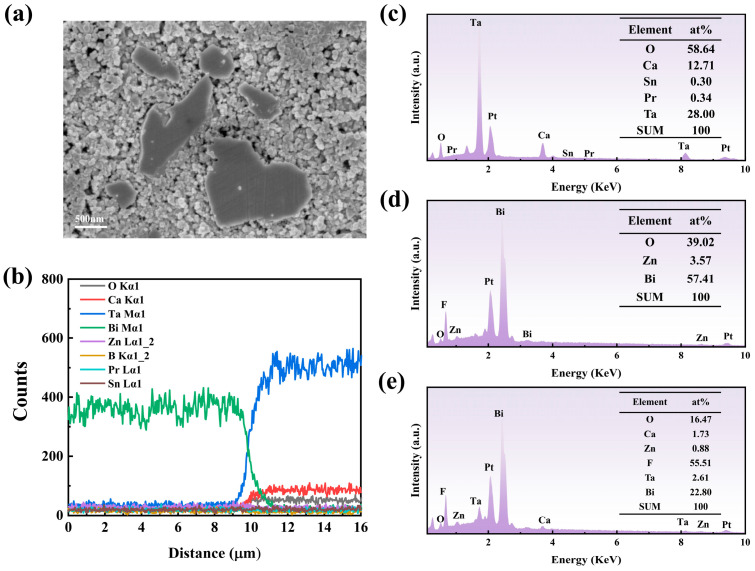
(**a**) SEM image showing the distribution of phosphor particles and the glass matrix; (**b**) EDS line analysis profile across a phosphor particle and the adjacent glass region; EDS point analysis results for the (**c**) phosphor particle, (**d**) glass matrix, and (**e**) interfacial region, respectively.

The morphology of the PiG sample was analyzed by SEM and EDS. The sample was treated with a 10 vol% HF solution for 5 s at first, and then Pt nanoparticles were sprayed on the surface for measurement. As shown in Figure 6a, the phosphor particles were embedded in the glass matrix and remained clearly distinguishable. The dark areas reflect the crystalline phase, while the bright areas reflect the remained glass phase after HF etching. The results of EDS line scanning crossing the interface between the phosphor particle and the glassy matrix are given in Figure 6b. Elements of Ta, Pr, and Sn were found to be concentrated in the phosphor particle region, while Bi and Zn were enriched in the glass region. This suggests that phosphor degradation and interfacial reactions were controlled during co-sintering.

EDS point analyses were further carried out in the phosphor particle region, the glass region, and their interface. The results are shown in Figure 6c–e, respectively. In the phosphor particle region, elements of O, Ca, Ta, Pr, and Sn dominated (Figure 6c), whereas O, Bi, and Zn were found in the glass region (Figure 6d). At their interface, all elements mentioned above were detected (Figure 6e), indicating that there should be an interfacial transition layer between the phosphor particle and the glass matrix. The high F content observed at the interface suggests that the region exhibits much weaker chemical stability against HF etching. It indicates that there was an undesirable interfacial reaction during PiG fabrication, which might result in photoluminescence quenching at high temperatures. Upon carefully checking the elemental distributions of Bi, Ca, and Ta found in Figure 6b, the above-mentioned phenomenon could be mainly ascribed to the mutual diffusion of Bi and Ca during PiG fabrication.

#### 3.2.2. Optical Properties

**Figure 7:**
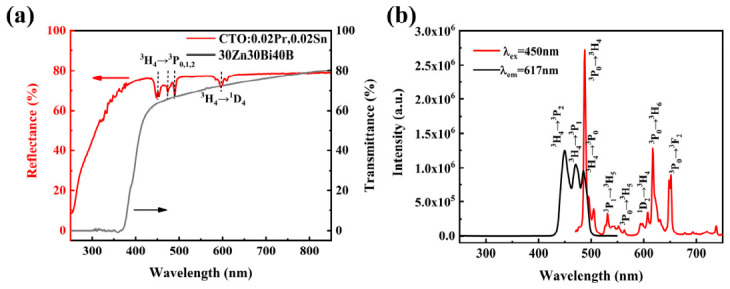
(**a**) Transmittance spectrum of the PG and diffuse reflectance spectrum of the CTO:0.02Pr, 0.02Sn phosphor; (**b**) excitation and emission spectra of the 30Zn30Bi40B-PiG sample.

Figure 7a shows the transmittance spectrum of the PG and the diffuse reflectance spectrum of the CTO:0.02Pr, 0.02Sn phosphor. The transmission window of the PG overlaps well with the characteristic absorption bands of Pr^3+^ ions in the phosphor, especially in the 450–500 nm range. This indicates that the visible-light excitation can be effectively transmitted through the glassy encapsulation and arrive at the surfaces of the phosphor particles. As shown in Figure 7b, the excitation bands and emission bands assigned to the characteristic transitions of Pr^3+^ ions were observed in the PiG sample, which were very similar to those found in the CTO:0.02Pr, 0.02Sn phosphor. No obvious peak position shifting in the PiG was found in comparison to the phosphor. However, the host-related broad-band absorption and the IVCT excitation band in the ultraviolet region are almost invisible. This is mainly due to the stronger UV absorption of the PG. On the whole, the PiG should be suitable for blue-light excitation.

#### 3.2.3. Temperature-Dependent Photoluminescence and Optical Thermometric Performance

**Figure 8:**
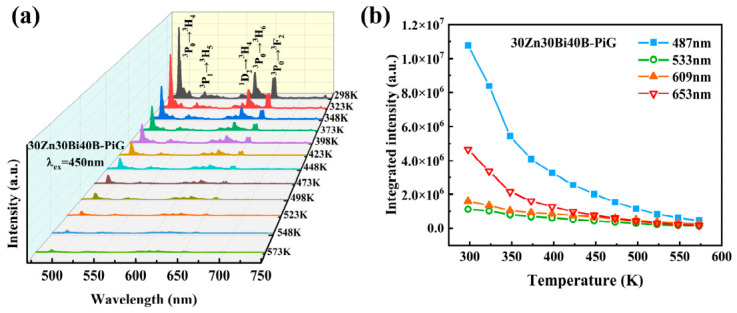
(**a**) Temperature-dependent emission spectra of 30Zn30Bi40B-PiG recorded from 298 to 573 K and (**b**) the corresponding integrated intensities of the characteristic emission bands.

Figure 8a shows the temperature-dependent emission spectra of the PiG sample under 450 nm excitation. It can be seen that the intensities of all characteristic emissions gradually decrease with increasing temperatures, indicating obvious thermal quenching during the heating process. The integrated intensities of the characteristic emission bands at 487 nm, 533 nm, 609 nm, and 653 nm as a function of the temperature are given in Figure 8b. They are in correspondence with the transitions of ^3^P_0_→^3^H_4_, ^3^P_1_→^3^H_5_, ^1^D_2_→^3^H_4_, and ^3^P_0_→^3^F_2_, respectively. In comparison with the phosphor, the emission intensity of the PiG dropped rapidly with the rising temperature. The CIE coordinates of the PiG sample at different temperatures are shown in Figure S4 and Table S3. This phenomenon might be ascribed to the phosphor/glass interface. Local structural distortion, coordination variation, and possible defect centers near the interface may serve as thermally activated nonradiative relaxation channels, thereby accelerating energy dissipation and leading to faster emission decay at elevated temperatures [33,34]. This indicates that the PiG might only be suitable for optical thermometry in low-to-moderate temperature ranges.

**Figure 9:**
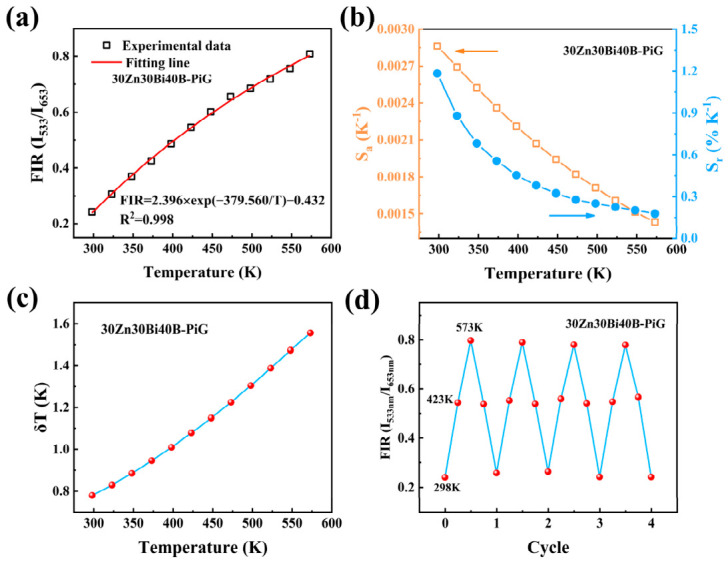
(**a**) FIR fitting curve; (**b**) the corresponding relative and absolute sensitivity curves; (**c**) temperature uncertainty (δ*T*) as a function of temperature; and (**d**) temperature cycling of FIR between 298 and 573 K in 30Zn30Bi40B-PiG.

Similarly to the CTO:0.02Pr, 0.02Sn phosphor, the optical thermometric performance of 30Zn30Bi40B-PiG was also evaluated, using the FIR based on the ^3^P_1_→^3^H_5_/^3^P_0_→^3^F_2_ transition pair, which shows the highest relative sensitivity among the investigated modes. As shown in Figure 9a,b, this FIR mode can be well fitted as a function of the temperature and exhibits maximum relative sensitivity of 1.18% K^−1^ at 298 K, which is slightly higher than that of the corresponding phosphor. The other two FIR modes are presented in Figure S5. The corresponding temperature uncertainty *δT* remains within 0.78–1.56 K over 298–573 K (Figure 9c), indicating acceptable temperature resolution. In addition, the heating–cooling cycle test (Figure 9d) gives reproducibility values of 98.9%, 99.0%, and 99.1% at 298, 423, and 573 K, respectively, confirming the good stability and repeatability of the FIR response. These results suggest that the thermometric mechanism governed by the combined effects of thermally coupled levels and IVCT-related nonradiative channels of Pr^3+^ remains in the PiG sample, although there is increased nonradiative relaxation due to the phosphor/glass interface.

As summarized in Table 1, the CaTa_2_O_6_:Pr^3+^ phosphor and the PiG based on it exhibit competitive thermometric performance in comparison with those reported in various Pr^3+^-activated luminescent thermometers. A relative sensitivity maximum of 1.09% K^−1^ at 298 K in the 298–573 K temperature range is found in the CTO:0.02Pr, 0.02Sn phosphor. The PiG sample achieves a relative sensitivity maximum of 1.18% K^−1^ at 298 K. These results indicate that CaTa_2_O_6_:Pr^3+^-based materials are promising candidates for luminescence thermometry.

## 4. Conclusions

The luminescence properties and optical temperature sensing performance of CaTa_2_O_6_:Pr^3+^ phosphors under blue-light excitation have been reported. The ICVT band is confirmed in the photoluminescence excitation spectrum. Under excitation at 450 nm, the optimal Pr^3+^ doping concentration is found to be 2 mol% and 0.7 mol% for the emissions from the ^3^P_J_ levels and the ^1^D_2_ level, respectively. Upon co-doping with Zr or Sn, the emission intensity is enhanced to 1.31 times and 1.34 times that in the Pr single-doped phosphor. Furthermore, a PiG composite was fabricated based on the CTO:0.02Pr, 0.02Sn phosphor, which exhibits a similar spectral profile regarding both the excitation and emission spectra to the phosphor. Employing the differences in the thermal-dependent photoluminescence spectra, an FIR mode based on ^3^P_1_→^3^H_5_/^3^P_0_→^3^F_2_ was found to be promising for optical temperature sensing. Relative sensitivity maxima of 1.09% K^−1^ and 1.18% K^−1^ at 298 K were found in the phosphor and the PiG under 450 nm excitation, respectively. CaTa_2_O_6_:Pr^3+^-based materials show potential for luminescence thermometry.

## Figures and Tables

**Table 1:** The maximum relative sensitivities of Pr^3+^-doped optical sensing materials.

Host Material	Transitions	Range (K)	*S*_r-Max_ (% K^−1^)	Ref.
NaCaY (MoO_4_)_3_	^3^P_1_→^3^H_5_/^3^P_0_→^3^F_2_	298–498	1.2 (298 K)	[35]
Ca_3_Y_2_Si_3_O_12_	^3^P_0_→^3^H_4_/P_1_→^3^H_5_	298–573	0.744 (298 K)	[36]
CaTiO_3_	^1^D_2_→^3^H_4_/^1^D_2_→^3^H_4_	20–200	0.82 (120 K)	[37]
Sr_3_Y_2_Ge_3_O_12_	^3^P_0_→^3^H_4_/^1^D_2_→^3^H_4_	13–1025	0.78 (188 K)	[38]
CaTa_2_O_6_ phosphor	^3^P_1_→^3^H_5_/^3^P_0_→^3^F_2_	298–573	1.09 (298 K)	This work
CaTa_2_O_6_ PiG	^3^P_1_→^3^H_5_/^3^P_0_→^3^F_2_	298–573	1.18 (298 K)	This work

## Data Availability

The original contributions presented in this study are included in the article/supplementary material. Further inquiries can be directed to the corresponding authors.

## Supplementary Materials

The following supporting information can be downloaded at https://www.mdpi.com/article/10.3390/ma19112324/s1. Figure S1: CIE chromaticity coordinates of CTO phosphors with different Pr^3+^ concentrations; Table S1: CIE chromaticity coordinates of CTO phosphors with different Pr^3+^ concentrations; Figure S2: CIE chromaticity coordinates of CTO:0.02Pr, 0.02Sn phosphor at different temperatures; Table S2: CIE chromaticity coordinates of CTO:0.02Pr, 0.02Sn phosphor at different temperatures; Figure S3: (a,c) FIR fitting curves and (b,d) the corresponding relative and absolute sensitivity curves of the CTO:0.02Pr, 0.02Sn phosphor. (a,b) ^3^P_1_→^3^H_5_/^3^P_0_→^3^H_4_; (c,d) ^1^D_2_→^3^H_4_/^3^P_0_→^3^H_4_; Figure S4: CIE chromaticity coordinates of PiG at different temperatures; Table S3: CIE chromaticity coordinates of PiG phosphor at different temperatures; Figure S5: (a,c) FIR fitting curves and (b,d) the corresponding relative and absolute sensitivity curves of the PiG. (a,b) ^3^P_1_→^3^H_5_/^3^P_0_→^3^H_4_; (c,d) ^1^D_2_→^3^H_4_/^3^P_0_→^3^H_4_.
