# Supplementary material for: Luminescent Properties and Optical Temperature Sensing Performance of CaTa2O6:Pr3+ Phosphors Under Blue-Light Excitation
Source: Materials (Basel). 2026 Jun 1;19(11):2324. doi: 10.3390/ma19112324 (PMC13258048; doi:10.3390/ma19112324)
Supplement: Supplementary file 1 [file materials-19-02324-s001.zip › materials-4299347-supplementary.pdf]

materials

MDPI

Supplementary Materials

Luminescent Properties and Optical Temperature Sensing Performance of $CaTa_2O_6:Pr^{3+}$ Phosphors Under Blue-Light Excitation

Quan Jiang [1,2], Jian Ruan [1,2,*], Chen Tian [2,3,*], Zijing Zhu [1,2], Shuang Zhang [1,2] and Chao Liu [1,2]

1. State Key Laboratory of Advanced Glass Materials, Wuhan University of Technology, Wuhan 430070, China; quanjiang_0382@whut.edu.cn (Q.J.); zijing@whut.edu.cn (Z.Z.); shuangzhang_22@whut.edu.cn (S.Z.); hite@whut.edu.cn (C.L.)
2. Research Center for Silicate Materials Engineering, Wuhan University of Technology, Wuhan 430070, China
3. State Key Laboratory of Silicate Materials for Architectures, Wuhan University of Technology, Wuhan 430070, China

* Correspondence: jian_ruan@whut.edu.cn (J.R.); tianchen@whut.edu.cn (C.T.)

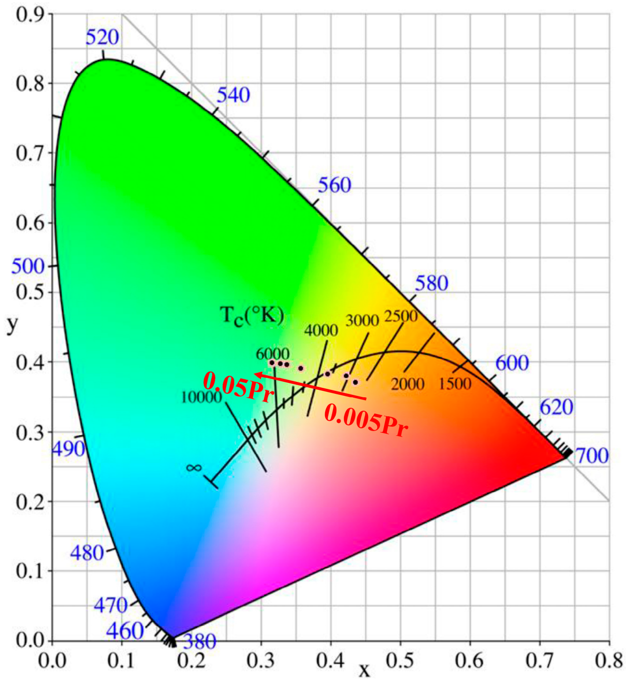

Figure S1. CIE chromaticity coordinates of CTO phosphors with different Pr^{3+} concentrations.

Table S1 CIE chromaticity coordinates of CTO phosphors with different Pr^{3+} concentrations.

Pr^{3+} Concentrations	CIE chromaticity coordinates (x, y)
0.005	(0.436, 0.370)
0.007	(0.422, 0.380)
0.01	(0.396, 0.382)
0.02	(0.358, 0.391)
0.03	(0.338, 0.396)
0.04	(0.329, 0.398)
0.05	(0.316, 0.399)

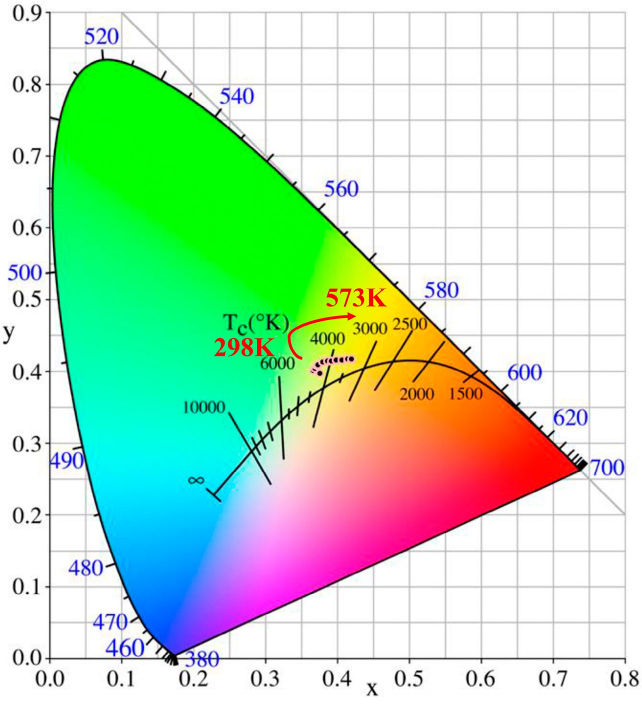

Figure S2. CIE chromaticity coordinates of CTO:0.02Pr, 0.02Sn phosphor at different temperatures.

Table S2 CIE chromaticity coordinates of CTO:0.02Pr, 0.02Sn phosphor at different temperatures.

Temperature (K)	CIE chromaticity coordinates (x, y)
298	(0.376, 0.397)
323	(0.367, 0.401)
348	(0.370, 0.404)
373	(0.373, 0.407)
398	(0.374, 0.409)
423	(0.380, 0.413)
448	(0.387, 0.414)
473	(0.392, 0.415)
498	(0.399, 0.416)
523	(0.407, 0.416)
548	(0.415, 0.417)
573	(0.420 0.418)

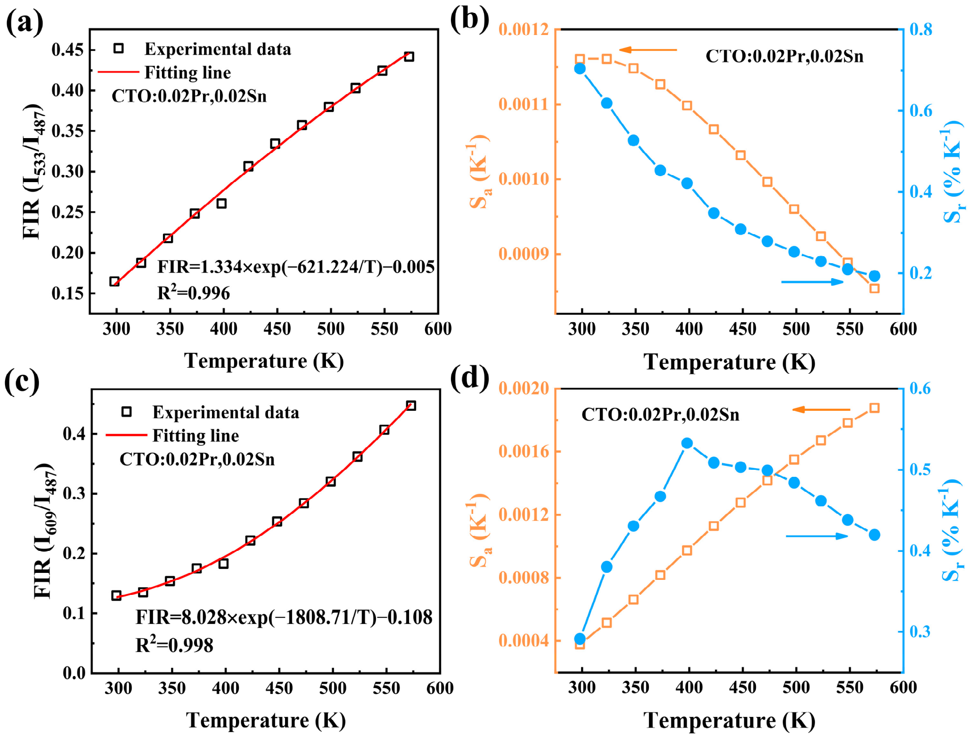

Figure S3. (a, c) FIR fitting curves and (b, d) the corresponding relative and absolute sensitivity curves of the CTO:0.02Pr, 0.02Sn phosphor. (a, b) $^3P_1 \rightarrow {}^3H_5/{}^3P_0 \rightarrow {}^3H_4$; (c, d) $^1D_2 \rightarrow {}^3H4/{}^3P_0 \rightarrow {}^3H_4$.
